# Tracing the Negative Results of Multiplex Real-Time PCR Assay for Diagnosis of Bacterial Pediatrics Meningitis

**DOI:** 10.1155/2023/3502666

**Published:** 2023-01-16

**Authors:** Abdollah Karimi, Sedigheh Rafiei Tabatabaei, Leila Azimi, Nasim Almasian Tehrani, Fatemeh Fallah, Iman Faghihian

**Affiliations:** ^1^Pediatric Infections Research Center, Research Institute for Children's Health, Shahid Beheshti University of Medical Sciences, Tehran, Iran; ^2^Department of Microbiology and Microbial Biotechnology, Faculty of Life Sciences and Biotechnology, Shahid Beheshti University, Tehran, Iran

## Abstract

The death because of meningitis remains high in some parts of the world. It is important to know the specific cause of meningitis because the treatment differs depending on the cause. This study aimed to trace the false-negative results of multiplex RT-PCR to detect *Streptococcus pneumoniae*, *Haemophilus influenzae*, and *Neisseria meningitidis* serogroup by two different molecular methods. In this study, the CSF of the suspicious pediatric for acute bacterial meningitis among children aged 1 month to 14 years who are admitted to the hospitals in four cities of a certain region of Iran was collected. *S. pneumoniae*, *H. influenzae*, and *N. meningitidis* in CSF samples were detected by single-tube multiplex RT-PCR and specific RT-PCR with a probe on the same specimens. In this cross-sectional study, 506 CSF samples were collected during one year. The multiplex RT-PCR can detect 3.3% and 2.2% of *S. pneumoniae* and *H. influenzae*, respectively. *N. meningitidis* was not detected. The CSF analysis was abnormal in 53% of 506 patients. On the other hand, 11.5%, 4.8%, and 4.1% of *S. pneumoniae*, *H. influenzae*, and *N. meningitidis* were identified, respectively, by specific RT-PCR assay, exactly on the same specimens. Various types of PCR can be used for pathogen identification. As we change the type of PCR in our study, we could approximately increase 15% our positive results and also consequently decrease our false-negative responses.

## 1. Introduction

CNS infections require rapid diagnosis, and this can prevent severe, long-term consequences or death [1]. Untreated bacterial meningitis can lead to a 50% mortality rate [2, 3]. Death can be occurred in 8-15% of untreated patients after 24 to 48 hours of onset symptom if early diagnosis and acceptable treatment have been not accrued [2, 3]. Moreover, 10–20% of the survivors suffered from the posteffects of meningitis such as disabilities and hearing loss [2, 3]. Bacterial meningitis caused by *N. meningitidis* is one of the most important IMD causes, affecting more than 500,000 people worldwide, resulting in approximately 50,000 deaths [2, 3]. The reported incidence of IMD varies by region, ranging from less than 0.5 cases per 100,000 in North America and less than one case per 100,000 in Europe to 10–1000 cases per 100,000 people during epidemic years in Africa [2]. Global surveillance of detected bacterial meningitides cases, including surveillance of the diversity of causative bacteria, is essential to managing disease and developing vaccines.

Various methods may be used to detect the agent causative of bacterial meningitides such as culture as a traditional method and also various types of the molecular assay such as conventional PCR, RT-PCR with SYBR™ Green PCR, and specific RT-PCR with probe [4]. Culture is the gold standard method to identify bacteria that cause meningitis. But the results of culture are negative mostly because of the use of antibiotics before catching the specimen [4]. Molecular detection can be more practical because of detecting the genome of microorganisms, and the viable bacteria are not necessary to detect. On the other hand, CSF culture has exposed limitations in determining bacterial meningitis diagnosis resulting from low bacterial titer in the CSF [5]. Specific RT-PCR with the probe is one of the most sensitive and specific molecular assays to detect microorganisms [6]. Multiplex RT-PCR is similarly an accurate method [7], but the use of multiple primers in a reaction can decrease the sensitivity of this method in comparison with nonmultiplex reactions. It is clear that the use of the probe increases the specificity of the detection method. This study uses as a reference a similar multisite facility-based meningitis surveillance study that is being conducted in Iran. The rationale to conduct this study is to generate better (reliable, laboratory-confirmed) data on the presence of acute pediatric bacterial meningitis, especially *N. meningitidis* cases in Iran to provide data to national decision-makers on the disease burden in the country and the effects of vaccination strategies. Moreover, this study aimed to describe the distribution of aetiological acute bacterial meningitis agents (*S. pneumoniae*, *H. influenzae* type B, and *N. meningitidis*) among collected meningitis cases by two different sets of RT-PCR, so that it will generate better data on detection of bacterial causative agent in meningitis in the country, and through the use of laboratory techniques, it helps improve the quality of surveillance and in-country diagnostic capacity.

## 2. Materials and Methods

This study was a hospital-based surveillance study to capture laboratory-confirmed cases of pediatric acute bacterial meningitis among children aged below 1 month to 14 years who are admitted to the hospitals in four cities in a certain region of Iran. They were 38% female and 62% male. In addition, it will describe the distribution of aetiological acute bacterial meningitis agents (*Streptococcus pneumoniae*, *Haemophilus influenzae* type B, and *Neisseria meningitidis*) in CSF samples among meningitis cases.

### 2.1. Samples and Setting

The multiplex real-time PCR and also specific RT-PCR with the probe were performed to detect *S. pneumoniae*, *H. influenzae* type B (Hib), and *N. meningitidis* in the CSF of the pediatrics. They suspected bacterial meningitis based on clinical signs and symptoms and also CSF analysis (for example, pleocytosis, high CSF protein, and low CSF glucose level).

### 2.2. Molecular Assays

Bacterial DNA was extracted from CSF samples directly, according to the manual of the specific DNA extraction kit (Qiagen Cat No./ID: 51304).

### 2.3. Multiplex RT-PCR

Single-tube, multiplex real-time PCR assay was performed for the simultaneous identification of bacterial agents. The specific gene targets are ctrA, bex, and ply for *N. meningitidis*, *H. influenzae* type B (Hib), and *S. pneumoniae*, respectively. The primers are shown in Table 1.

### 2.4. Specific RT-PCR

The specific RT-PCR with the primers and probes (Table 2) was prepared in a separate tube for each bacterium. In this method, just *H. influenzae* was identified not Hib.

## 3. Results

At first, the multiplex RT-PCR could detect 9 (3.1%) and 6 (2.1%) *S. pneumoniae* and *H. influenzae*, respectively. *N. meningitidis* was not detected in any of the samples. The results of CSF analysis were abnormal in 281 (56%) of 506 patients. After that, by using a specific RT-PCR assay with the probe, 30 (10.6%), 13 (4.6%), and 10 (3.6%) of *S. pneumoniae*, *H. influenzae*, and *N. meningitidis*, respectively, were identified (Table 3).

## 4. Discussion

Meningitis can be caused by bacterial, viral, or fungal agents that invade the cerebrospinal fluid (CSF), although the highest global burden is related to bacterial meningitis and can cause morbidity and mortality throughout the world [4, 10, 11]. On the other hand, meningococcal meningitis is mainly observed in preschool children and young people, according to the WHO report [4, 10]. Bacterial meningitis occurs mostly in childhood, and the aetiological pathogens can be diverse in different age groups of children [3, 12, 13]. According to the other studies, the microbial cause of the meningitis may vary based on the time of study, the geographical area, and also the age of patients [3, 12, 13]. *H. influenzae* is involved in bacterial meningitis especially in children less than 5 years old [14].

A systematic review and meta-analysis on the worldwide etiology of bacterial meningitis in 2018 was performed by Anouk et al. They showed that the most causative prevalent pathogens were *N. meningitidis* and *S. pneumoniae* in bacterial meningitis cases in all age groups and *S. pneumoniae* in children [3]. Our results are similar to those of Anouk et al. because the most causative pathogen in children with bacterial meningitis was *S. pneumoniae*.

Moreover, a meta-analysis has been done among patients with bacterial meningitis in Iran. The results of this study demonstrated that pneumococcal meningitis can be one of the main public health problems in Iran [15].

Wagner et al. reported a high agreement (99%) between culture and multiplex RT-PCR for bacterial identification [16]. They can detect bacterial agents in four culture-negative samples by using multiplex RT-PCR. It is notable that we presume the high rate of false-negative results in culture because the collected CSF samples are done after beginning the antibiotic therapy. So, we expect a more positive result in the PCR assay. On the other hand, more positive results could find if we use a suitable molecular method. As our results showed, when we changed our molecular method and improved it, we could detect more bacterial agents in CSF samples. The results of the study done by Haddad-Boubaker et al. showed that the sensitivity of the specific real-time PCR was up to 67.10^−4^ ng/*μ*L DNA detection and the specificity was 100% [5].

Kwambana-Adams et al. worked on 35 invasive agents in collected CSF specimens from children younger than 5 years with assumed meningitis by probe-based triplex real-time PCR across West Africa [17]. They can detect 0.6% *S. pneumoniae*, 1% *N. meningitidis*, and 0.2% *H. influenzae*. Our results showed 13% *S. pneumoniae*, 5.5% *H. influenzae*, and 4.1% *N. meningitidis* detection. The lower frequency of selected bacteria in this study compare with us can because the use vaccine in Africa as a country in global meningitis belt. It can be concluded that more bacterial agents could be detected in West Africa if a single specific RT-PCR with the probe was used because we could detect more bacterial pathogen when changing our method from multiplex RT-PCR to single RT-PCR.

## 5. Conclusion

Detection of a causative of meningitis can be helpful to choose the best therapeutic process as soon as possible. In this regard, selecting the most accurate and rapid method to identify relevant agents is necessary. The molecular assay is a rapid and reliable test in this way, but it is notable that several molecular methods and various types of PCR can be used to detect the cause of meningitis. As we change the type of real-time PCR in our study, we could achieve more positive results and also consequently decrease our false-negative responses. For example, we get more positive results of *S. pneumoniae*, *H. influenzae,* and *N. meningitidis* when choosing the more accurate molecular method. It is important to know the specific cause of meningitis because the treatment differs depending on the cause.

## Figures and Tables

**Table 1:** Primer sequencing in multiplex real-time PCR.

Bacteria	Gene	Primer sequencing (5′ to 3′)	Nucleotides target	Reference
*N. meningitidis*	CTR-F	GCTGCGGTAGGTGGTTCAA	617–635	[8]
	CTR-R	TTGTCGCGGATTTGCAACTA	727–708	

Hib	bexA-F	GGCGAAATGGTGCTGGTAA	142–160	[8]
	bexA-R	GGCCAAGAGATACTCATAGAACGTT	241–217	

*S. pneumoniae*	Ply-F	TGCAGAGCGTCCTTTGGTCTAT	849–915	[8]
	Ply-R	CTCTTACTCGTGGTTTCCAACTTGA	974–950	

**Table 2:** Primer sequencing of specific real-time PCR.

Bacteria	Primers	Primers and probe sequence	Explanations	References
*N. meningitidis*	sodC-F	GCACACTTAGGTGATTTACCTGCAT	—	[9]
	sodC-R	CCACCCGTGTGGATCATAATAGA		
	sodC-P	CATGATGGCACAGCAACAAATCCTGTTT	5′ FAM, 3′ BHQ1	

*H. influenzae*	Hpd-F	GGTTAAATATGCCGATGGTGTTG	—	[9]
	Hpd-R	TGCATCTTTACGCACGGTGTA		
	Hpd-P	TTGTGTACACTCCGT“T”GGTAAAAGAACTTGCAC	5′ FAM, BHQ1 on “T,” 3′ SpC6	

*S. pneumoniae*	lytA-F	ACGCAATCTAGCAGATGAAGCA	—	[9]
	lytA-R	TCGTGCGTTTTAATTCCAGCT		
	lytA-P	TGCCGAAAACGC“T”TGATACAGGGAG	5′ FAM, BHQ1 on “T,” 3′ SpC6	

**Table 3:** Demographic data of positive for PCR patients.

Detected bacteria	Age	Sex (%)		CSF analysis
		Male	Female	
*S. pneumoniae*	One month to 12 years old	70	30	Abnormal
*H. influenzae*	Below one month to 10 years old	77	23	Abnormal
*N. meningitidis*	Below one month to 14 years old	70	30	Abnormal

## Data Availability

The data used to support the findings of this study are included within the article.

## Abbreviations

RT-PCR: Real-time polymerase chain reaction
CSF: Cerebrospinal fluid
CNS: Central nervous system
IMD: Invasive meningococcal diseases.
